# Attitudes toward smart sports technologies and artificial intelligence: an examination from cognitive, affective and behavioral dimensions

**DOI:** 10.3389/fpubh.2026.1830510

**Published:** 2026-05-14

**Authors:** Irfan Kara, Yunus Sahinler, Mustafa Can Koc, Mustafa Yasar Sahin, Kenan Sebin, Laurentiu-Gabriel Talaghir, Teodora Mihaela Iconomescu, Liliana Nanu, Ștefan Cristian Liușnea, Gabriel Marian Manolache

**Affiliations:** 1 Faculty of Sports Sciences, Istanbul Gelisim University, Istanbul, Türkiye; 2 Faculty of Sports Sciences, Gazi University, Ankara, Türkiye; 3 Faculty of Sports Sciences, Atatürk University, Erzurum, Türkiye; 4 Faculty of Physical Education and Sport, Dunarea de Jos University of Galati, Galaţi, Romania

**Keywords:** artificial intelligence, attitude, smart sports technologies, sports sciences, wearable technologies

## Abstract

**Background:**

The aim of this study is to examine university students' attitudes toward smart sports technologies and artificial intelligence within the framework of cognitive, affective, and behavioral dimensions, and to determine the predictive and mediating effects of these dimensions on attitudes toward wearable technological sports products.

**Objective:**

A total of 684 University Students' studying in the Faculties of Sports Sciences of universities in Istanbul participated in the study.

**Methods:**

The data collection tools used were a Personal Information Form, an Attitude Scale Toward Wearable Technological Sports Products, and an Attitude Scale Toward Artificial Intelligence. IBM SPSS Statistics 27.0 and Hayes PROCESS Macro were used for data analysis; descriptive statistics, independent samples *t*-test, Pearson correlation analysis, multiple linear regression analysis, and mediation analysis were applied.

**Results:**

The findings revealed significant gender differences in attitudes toward smart sports technologies and artificial intelligence, as well as significant and positive relationships among cognitive, affective, and behavioral attitudes and a strong predictive effect of these attitudes on attitudes toward wearable sports technologies.

**Conclusions:**

In conclusion, it can be said that individuals' cognitive evaluations of artificial intelligence influence their emotional and behavioral tendencies, thus shaping their attitudes toward smart sports technologies. These findings demonstrate that psychological attitude dimensions play a significant role in the adoption of artificial intelligence and wearable technologies in sports environments.

## 1 Introduction

The rapid advancement of digital technologies has led to a profound transformation across multiple domains, including sports science, where traditional approaches are increasingly being replaced by data driven and technology supported practices. This transformation is closely associated with the broader concept of digitalization, which emphasizes the integration of advanced computational tools into human performance contexts (1, 2). In recent years, the increasing availability of large scale data and developments in computational power have enabled more sophisticated monitoring and evaluation of athlete performance and health indicators (3). Furthermore, the growing emphasis on evidence based practice in sports science has accelerated the adoption of digital tools that support objective and systematic decision making processes (4). In this context, emerging technologies have begun to reshape how performance is analyzed, interpreted, and enhanced in both individual and team sports.

The digital transformation process has fundamentally changed many applications in the field of sports science, such as performance analysis, training planning, injury prevention, and decision support systems. In particular, the integration of artificial intelligence (AI), big data analytics, and smart sensor technologies has accelerated data driven decision making processes in sports environments and made performance management more precise and predictable. These technologies play a critical role not only in terms of performance optimization but also in the physiological monitoring of athletes, tactical analysis, and the development of personalized training programs (5, 6).

AI-based sports technologies enable multidimensional analysis of sports performance through tools such as machine learning, computer vision, wearable sensors, and predictive modeling. Thanks to these technologies, athletes' movement patterns, load levels, and performance trends can be analyzed in real time, and training processes can be optimized based on scientific data (7). Especially in professional sports environments, AI systems are increasingly used as decision support mechanisms in areas such as performance prediction and injury risk forecasting (6).

However, the proliferation of sports technologies is not only a technical and performance oriented transformation; it has also made individuals' perceptions and attitudes toward these technologies significant. Individuals' cognitive evaluations, emotional responses, and behavioral tendencies play a decisive role in the adoption process of technological innovations. This is supported by theoretical approaches that explain technology acceptance and usage behavior. The Technology Acceptance Model reveals that perceived utility and ease of use are decisive factors in individuals' adoption of technology, demonstrating that cognitive evaluations guide behavior toward technology (8). In the adoption process of wearable technologies, perceived utility, ease of use, and behavioral intention are also cited as key determinants of technology acceptance (9, 10).

In the psychology literature, the concept of attitude is considered a multidimensional structure consisting of cognitive, affective, and behavioral components (11). In this context, examining attitudes toward intelligent sports technologies requires a holistic understanding of individuals' cognitive evaluations of the functionality of these systems, their emotional responses to the technology, and their behavioral tendencies regarding their intention to use it. Attitudes toward AI-powered systems are considered a critical determinant for the effective application and sustainable use of the technology, particularly for athletes, coaches, and sports professionals.

Although recent years have extensively focused the effects of artificial intelligence and smart systems on performance in the sports science literature, studies addressing the psychological dimensions of individual attitudes toward these technologies appear to be limited. However, the successful implementation of technological innovations depends not only on the technical infrastructure but also on the users' level of acceptance and psychosocial adaptation to the technology. Therefore, examining attitudes toward smart sports technologies based on cognitive, affective, and behavioral dimensions constitutes an important research area for understanding technology integration in sports environments. It is stated that the acceptance of artificial intelligence and wearable technologies is directly related to individuals' attitudes toward technology, perceived benefits, and intentions to use them (12).

In this context, the present study aims to examine individuals' mental evaluations, emotional approaches, and behavioral tendencies regarding smart sports technologies and artificial intelligence applications from a multidimensional perspective. Thus, it aims to contribute to the literature on the psychological dimensions of technological transformation in sports sciences. Accordingly, the following hypotheses have been developed to determine the relationships and predictive mechanisms between cognitive, affective, and behavioral attitudes toward artificial intelligence and attitudes toward wearable technological sports products:

*Research Hypotheses*

*H1: There are positive and significant relationships between cognitive, affective, and behavioral attitude dimensions toward artificial intelligence and positive attitudes toward wearable technological sports products*.
*H2: Cognitive, affective, and behavioral attitude dimensions toward artificial intelligence positively and significantly predict positive attitudes toward wearable technological sports products*.
*H3: Affective and behavioral attitude dimensions play a mediating role in the effect of cognitive attitude toward artificial intelligence on positive attitudes toward wearable technological sports products*.

## 2 Materials and methods

### 2.1 Population and sample

**Figure 1:**
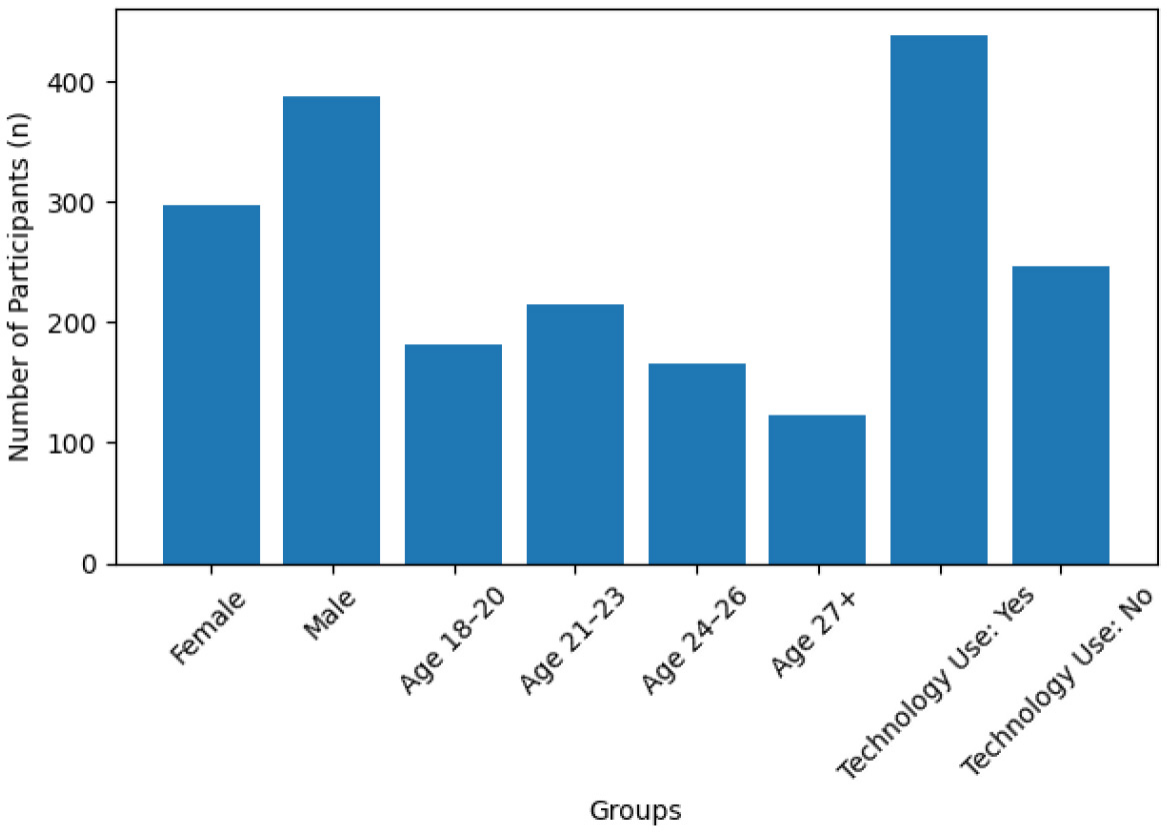
Distribution of participants by gender, age group, and smart sports technology use.

A total of 684 University Students' participated in the study. Demographic characteristics revealed that 56.6% of the sample was male (*n* = 387) and 43.4% was female (*n* = 297). Regarding age groups, the largest group was 21–23 years old (31.3%, *n* = 214), followed by 18–20 years old (26.6%, *n* = 182), 24–26 years old (24.1%, *n* = 165), and 27 years and older (18.0%, *n* = 123). When evaluated based on smart sports technology usage, a significant majority of participants used these technologies (64.0%, *n* = 438), while 36.0% (*n* = 246) did not (Figure 1).

### 2.2 Data collection tools

In this section, the personal information form created by the researcher, the Wearable Technological Sports Products Attitude Scale (WTSPTS), and the University Students' Attitude Toward Artificial Intelligence (SATAI) Scale will be used.

### 2.3 Personal information form

A Personal Information Form, created by the researchers, was used to determine the demographic characteristics of the participants in the study. This form included basic information such as the participants' gender, age, and usage status of smart sports technologies.

#### 2.3.1 Wearable technological sports products attitude scale (WTSSFTS)

To determine students' attitudes toward wearable technological sports products, a 14-item scale with 2 sub-dimensions, positive attitude and negative attitude, developed by Çar et al. (13) was used. The scale is a 5-point Likert type scale ranging from 1-Strongly Disagree to 5-Strongly Agree. Items 10-11-12-13-14 in the scale were reverse coded. The Cronbach Alpha value of the scale was calculated as 0.841.

#### 2.3.2 University students' attitudes toward artificial intelligence (SATAI) scale

In this study, the university students' attitudes toward artificial intelligence scale (SATAI) was used to determine individuals' cognitive, affective, and behavioral tendencies toward artificial intelligence. The scale was first developed by Suh and Ahn (14) and aims to measure students' general attitudes toward artificial intelligence. The Turkish adaptation, validity, and reliability studies were carried out by Turgut and Kunuroglu (15). The scale consists of 26 items and is answered using a 5-point Likert-type rating system (1 = Strongly Disagree, 5 = Strongly Agree). The lowest possible score on the scale is 26, and the highest is 130, with higher scores indicating more positive attitudes toward artificial intelligence. There is no reverse coded items in the scale. The scale was found to consist of three sub-dimensions: Cognitive dimension: Items 1, 2, 3, and 4; Affective dimension: Items 5–14; Behavioral dimension: Items 15–26. These three dimensions allow for a holistic assessment of how individuals think (cognitive), feel (affective), and behave (behavioral) toward artificial intelligence. The scale is a reliable measurement tool for understanding university students' awareness, interest, and usage tendencies toward artificial intelligence. The Cronbach Alpha value of the scale was calculated as 0.837.

### 2.4 Research model

This research was designed using a correlational survey model within the scope of quantitative research methods. According to Karasar (16), the correlational survey model is a research approach that aims to determine the degree of covariation between two or more variables.

### 2.5 Analysis of data

The data obtained from the research were analyzed using the IBM SPSS Statistics 27.0 software package. Before analysis, the dataset was examined for missing data, outliers, and normality assumptions. The suitability of the variables for normal distribution was evaluated using skewness and kurtosis values, and it was determined that all values were within the ±1 range. The internal consistency levels of the scales were evaluated using Cronbach's alpha coefficient. Descriptive statistics (mean, standard deviation, minimum and maximum values) were calculated to determine the attitude levels of the participants toward smart sports technologies and artificial intelligence. An independent samples *t*-test was applied to compare attitude scores according to gender. Pearson product moment correlation analysis was performed to determine the relationships between the variables, and a correlation heatmap graph was created to visually present the relationships. Multiple linear regression analysis was applied to determine the predictive level of cognitive, affective, and behavioral dimensions of artificial intelligence attitudes toward wearable technological sports products. In addition, Model 4 mediation analysis was performed using the Hayes PROCESS Macro (Version 4.2) to examine the indirect effects between variables. The significance of indirect effects was evaluated using a 5,000-member bootstrap sample and a 95% confidence interval. To assess the adequacy of the sample size, a *post hoc* power analysis was performed using the *G* ^*^ Power 3.1 program, and it was determined that the current sample size had sufficient statistical power to detect moderate effect sizes (*f*^2^ = 0.15) (power > 0.99). In all analyses, the statistical significance level was accepted as *p* < 0.05.

## 3 Result

Table 1 presents the descriptive statistics, reliability coefficients, and distribution characteristics of the sub-dimensions of the Attitude Scale Toward Smart Sports Technologies and Artificial Intelligence. First, examining the arithmetic mean values of the sub-dimensions of the scale, it is seen that participants have the highest mean in the cognitive dimension (*M* = 3.78, SD = 0.71). This indicates that participants have a relatively high level of knowledge and awareness about smart sports technologies and artificial intelligence. The cognitive dimension is followed by positive attitude (*M* = 3.72, SD = 0.69), affective dimension (*M* = 3.65, SD = 0.74), and behavioral dimension (*M* = 3.59, SD = 0.76), respectively. These results show that participants exhibit a positive inclination toward technology, both emotionally and behaviorally. In contrast, the mean of the negative attitude dimension was found to be lower (*M* = 2.41, SD = 0.81). This finding indicates that participants' negative views toward smart sports technologies are relatively limited.

**Table 1:** Descriptive statistics and reliability coefficients of attitude scales toward smart sports technologies and artificial intelligence (*N* = 684).

Variable	*N*	*M*	SD	Cronbach	Skewness	Kurtosis
Cognitive	684	3.78	0.71	0.82	−0.41	−0.32
Affective	684	3.65	0.74	0.86	−0.36	−0.28
Behavioral	684	3.59	0.76	0.88	−0.29	−0.21
WTSSFTS positive attitude	684	3.72	0.69	0.84	−0.44	−0.37
WTSSFTS negative attitude	684	2.41	0.81	0.80	0.52	0.48

When the internal consistency coefficients (Cronbach's α) of the scale were examined, α =0.82 was calculated for the cognitive dimension, α = 0.86 for the affective dimension, α = 0.88 for the behavioral dimension, α =0.84 for the positive attitude dimension, and α = 0.80 for the negative attitude dimension. These values show that all sub-dimensions of the scale have a high level of reliability and that the measurements are consistent. The assumption of normal distribution of the data was also evaluated by examining the skewness and kurtosis values. Skewness values ranged from −0.44 to 0.52, and kurtosis values ranged from −0.37 to 0.48. These values being within the ±1 range indicates that the data are close to a normal distribution.

Table 2 shows that an independent samples *t*-test was applied to determine whether attitudes toward smart sports technologies and artificial intelligence differed according to gender. According to the analysis results, the mean scores of male participants were significantly higher than those of female participants in the cognitive (*t* = −2.94, *p* =0.003), affective (*t* = −2.48, *p* = 0.013), behavioral (*t* = −3.21, *p* = 0.001), and positive attitude (*t* = −2.86, *p* = 0.004) dimensions. Conversely, the mean score of female participants was higher than that of male participants in the negative attitude dimension, and this difference was statistically significant (*t* = 2.22, *p* = 0.027). These findings indicate that attitudes toward smart sports technologies and artificial intelligence differ significantly according to gender (Table 2).

**Table 2:** Comparison of attitude scores toward smart sports technologies and artificial intelligence by gender (independent samples *t*-test).

Variable	Female (*n* = 297) M ±SD	Male (*n* = 387) M ±SD	*t*	*p*
Cognitive	3.69 ± 0.73	3.85 ± 0.69	−2.94	**0.003** ^*^
Affective	3.57 ± 0.76	3.71 ± 0.72	−2.48	**0.013** ^*^
Behavioral	3.48 ± 0.78	3.67 ± 0.74	−3.21	**0.001** ^*^
WTSSFTS positive attitude	3.64 ± 0.71	3.79 ± 0.67	−2.86	**0.004** ^*^
WTSSFTS negative attitude	2.49 ± 0.83	2.35 ± 0.79	2.22	**0.027** ^*^

^*^A significant difference is expressed. Significant difference indicator in bold.

**Figure 2:**
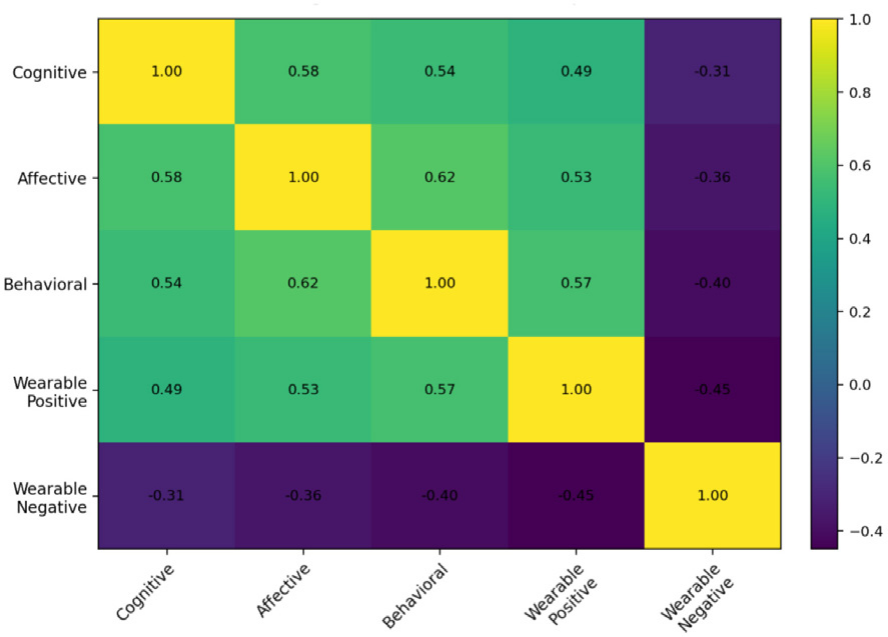
Pearson correlation coefficients between attitude dimensions toward smart sports technologies and artificial intelligence (*N* = 684).

Figure 2 illustrates the correlation heatmap showing the relationships among cognitive, affective, behavioral attitudes toward artificial intelligence and attitudes toward wearable sports technology products. The results indicate moderate and positive correlations among the cognitive, affective, and behavioral dimensions (*r* = 0.54–0.62), suggesting that individuals who evaluate artificial intelligence positively at the cognitive level also tend to develop positive emotional and behavioral tendencies. Positive attitudes toward wearable sports technology was moderately and positively correlated with cognitive (*r* = 0.49), affective (*r* = 0.53), and behavioral attitudes (*r* = 0.57), indicating that favorable perceptions of artificial intelligence are associated with stronger acceptance of wearable sports technologies. Conversely, negative attitudes toward wearable sports technology showed moderate negative correlations with cognitive (*r* = −0.31), affective (*r* = −0.36), and behavioral attitudes (*r* = −0.40), suggesting that more positive artificial intelligence attitudes are associated with lower resistance toward wearable sports technologies.

Multiple linear regression analysis was performed to determine the predictive level of artificial intelligence attitude dimensions toward positive attitudes toward wearable technological sports products. The analysis results show that the model is statistically significant [*F* (3, 680) = 151.72, *p* < 0.001] and it was determined that the model explained 40% of the total variance (*R*^2^ = 0.40). Cognitive attitude was found to significantly predict positive attitudes toward wearable sports technologies (β = 0.28, *p* < 0.001). Similarly, affective attitude was also determined to be a significant and positive predictor (β = 0.31, *p* < 0.001). Behavioral attitude was found to be the strongest predictor variable, and this variable was found to have a significant and strong effect on positive attitude (β = 0.36, *p* < 0.001).

To examine the indirect effects of cognitive attitude toward artificial intelligence on positive attitude toward wearable sports technologies, mediation analysis was performed using the Hayes PROCESS Macro Model 4. Results obtained using the Bootstrap method (5,000 samples) showed that the indirect effect of cognitive attitude on positive attitude was significant [β =0.36, 95% CI (0.28, 0.44)]. It was determined that cognitive attitude significantly influenced positive attitude through affective attitude [β = 0.12, 95% CI (0.08, 0.17)]. Similarly, behavioral attitude was found to play a significant mediating role [β = 0.15, 95% CI (0.10, 0.21)]. Furthermore, the path cognitive → affective → behavioral → positive attitude was also found to be significant [β = 0.09, 95% CI (0.05, 0.14)] (Table 3).

**Table 3:** Indirect effects of cognitive, affective, and behavioral dimensions of attitude toward artificial intelligence on attitudes toward wearable sports technology (PROCESS model 4, bootstrap = 5,000, *N* = 684).

Path	Effect (β)	Boot SE	Below 95% CI	95% GA and above
Cognitive → Affective → Positive attitude	0.12	0.02	0.08	0.17
Cognitive → Behavioral → Positive attitude	0.15	0.03	0.10	0.21
Cognitive → Affective → Behavioral → Positive attitude	0.09	0.02	0.05	0.14
Total indirect effect	0.36	0.04	0.28	0.44

Direct Effect: β = 0.21, SE = 0.04, *p* < 0.01.

Bootstrap sample size = 5,000. GA = confidence Interval.

**Figure 3:**
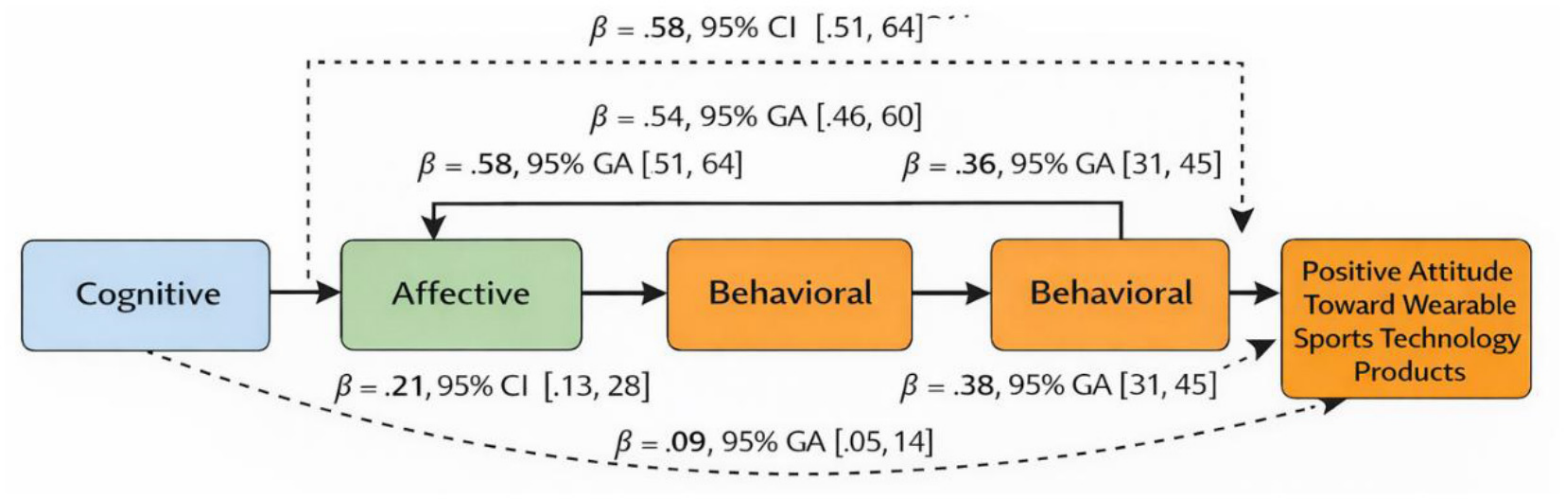
Mediation model illustrating the effects of cognitive attitude on positive attitudes toward wearable sports technology products through affective and behavioral attitudes. Standardized regression coefficients (β) and 95% confidence intervals are presented. All paths shown are statistically significant (*p* = <0.05).

Figure 3 presents the mediation model examining the relationships between cognitive, affective, and behavioral attitudes toward artificial intelligence and positive attitudes toward wearable sports technology products. The results indicate that cognitive attitude significantly predicted affective attitude [β = 0.58, 95% CI (0.51, 0.64)] and behavioral attitude [β = 0.54, 95% CI (0.46, 0.60)]. In turn, affective attitude significantly predicted behavioral attitude [β = 0.58, 95% CI (0.51, 0.64)]. Behavioral attitude was found to be the strongest direct predictor of positive attitude toward wearable sports technology products [β = 0.38, 95% CI (0.31, 0.45)], followed by affective attitude [β = 0.36, 95% CI (0.31, 0.45)]. Additionally, cognitive attitude had a significant direct effect on positive attitude [β = 0.21, 95% CI (0.13, 0.28)]. The indirect effect of cognitive attitude on positive attitude through affective and behavioral attitudes was also significant [β = 0.09, 95% CI (0.05, 0.14)], indicating a partial mediation effect (Table 4).

**Table 4:** Multiple linear regression analysis of the prediction of positive attitudes toward wearable technological sports products by artificial intelligence attitude dimensions (*N* = 684).

Predictor Variable	B	SE	β	*t*	*p*
Cognitive	0.29	0.04	0.28	6.84	**0.001** ^***^
Affective	0.34	0.05	0.31	7.42	**0.001** ^***^
Behavioral	0.38	0.05	0.36	8.15	**0.001** ^***^

Model Summary: *R* = 0.63, *R*^2^ = 0.40, *F*(3, 680) = 151.72, *p* < 0.01.

*B* = Unstandardized coefficient; β = Standardized coefficient.

^***^p < 0.01.

Significant difference indicator in bold.

## 4 Discussion

The aim of this research is to examine the effects of sports science students' cognitive, affective, and behavioral attitudes toward artificial intelligence on their attitudes toward wearable sports technologies. The sample size shows parallels when compared to similar studies (17, 18). The findings showed that participants generally had moderate to high levels of positive attitudes toward both artificial intelligence and wearable sports technologies. This result indicates that the increasing use of digital technologies and data-driven performance analysis systems in sports environments positively influences individuals' perceptions and attitudes toward these technologies. Indeed, it is stated that artificial intelligence and wearable sensor technologies provide significant advantages in areas such as monitoring sports performance, load management, and reducing injury risk (6, 7, 17, 29). In this context, the development of positive attitudes toward these technologies by sports science students can be considered an important reflection of the digital transformation process in sports. Supporting this finding, recent studies emphasize that the integration of digital technologies into sports education enhances students' technological awareness and facilitates their adaptation to innovative performance tools (19, 20). It is also reported that AI-powered wearable technologies contribute to the planning of training processes in a more objective, individualized, and scientifically based manner by analyzing athletes' physiological and performance data in real time (17, 18). Furthermore, similar research suggests that individuals who are more familiar with digital systems tend to develop more favorable attitudes toward emerging sports technologies, which in turn accelerates technology adoption in sports contexts (21).

One of the key findings of the study is the existence of significant and positive relationships between cognitive, affective, and behavioral attitudes toward artificial intelligence. This result is consistent with the core assumptions of the Technology Acceptance Model, which suggests that individuals' perception of a technology as useful and functional reinforces their emotional responses and usage behaviors toward that technology (8). Indeed, extended models of technology acceptance show that individuals' cognitive evaluations have direct and significant effects on attitudes toward technology and behavioral intention to use it (22). In addition, the Unified Theory of Acceptance and Use of Technology highlights that factors such as performance expectancy and effort expectancy play a crucial role in shaping users' behavioral intentions (22). Similarly, it is stated that attitudes toward wearable technologies and intention to use them are among the determining psychological factors in the adoption of these technologies, and that users' positive perceptions of the technology significantly increase their usage behavior (9). Consistent with these findings, recent studies in sports technology contexts report that positive cognitive and affective evaluations of AI systems significantly predict athletes' and students' willingness to engage with wearable technologies (23). Therefore, the strong relationships identified in this study can be interpreted as an indication that multidimensional attitudes toward artificial intelligence play a decisive role in the acceptance and effective use of wearable sports technologies.

More recent research also reveals that cognitive perceptions play a critical role in the adoption of artificial intelligence technologies, and that individuals' positive evaluations of the benefits of the technology significantly strengthen emotional acceptance and behavioral usage tendencies toward the technology (23, 24). In this context, it can be considered that increasing cognitive awareness of artificial intelligence is an important factor that can increase individuals' development of positive emotional attitudes toward these technologies and their usage behaviors. Another important finding of the study is that cognitive, affective, and behavioral attitude dimensions toward artificial intelligence significantly and positively predict attitudes toward wearable sports technologies. In particular, the emergence of behavioral attitude as the strongest predictor variable shows that individuals' readiness levels and intentions to use the technology play a critical role in the technology adoption process. This result parallels previous studies that have shown that behavioral intention is one of the strongest determinants of technology use in the adoption of wearable technologies (10). The literature emphasizes that factors such as perceived benefit, ease of use, and behavioral intention are key variables determining technology acceptance in wearable sports technologies (24, 25). However, it is reported that the scientific and practical advantages provided by wearable technologies used in sports environments, such as performance analysis, load tracking, and training optimization, play a significant role in individuals developing positive attitudes toward these technologies (6, 17). Similarly, it is stated that individuals' behavioral readiness and intention to use the technology are among the strongest psychological determinants in the adoption of wearable technologies (25, 26).

When these findings are considered together, it can be said that positive attitudes toward artificial intelligence constitute an important psychological basis that increases individuals' tendency to adopt wearable sports technologies.

Mediation analysis findings revealed that affective and behavioral attitude dimensions play a partial mediating role in the effect of cognitive attitude toward artificial intelligence on attitudes toward wearable sports technologies. This finding shows that technology acceptance is not only a process based on cognitive evaluations, but also that individuals' emotional responses and behavioral tendencies toward technology are important components of this process. Attitude theory, which argues that attitude consists of cognitive, affective, and behavioral components, emphasizes that these dimensions form a holistic and dynamic structure that mutually influences each other (11). Similarly, studies on the adoption of artificial intelligence technologies show that individuals' emotional trust levels and usage intentions toward technology directly and indirectly affect technology acceptance (27). In this context, increased cognitive awareness of artificial intelligence among individuals may create a psychological mechanism that facilitates the adoption of wearable sports technologies by strengthening positive emotional evaluations and intentions to use the technology. This finding supports multidimensional theoretical models of technology acceptance, demonstrating that positive cognitive evaluations of artificial intelligence shape overall attitudes toward the technology through emotional and behavioral processes.

The research findings offer significant practical implications for sports science education and practices. Implementing structured training programs on artificial intelligence and wearable sports technologies can enhance individuals' cognitive awareness of these technologies, fostering positive attitudes toward them and promoting technology acceptance. It is believed that technology based training and applications, particularly for athletes, coaches, and sports science students, can contribute to the more effective and conscious use of AI-supported systems in performance analysis, workload tracking, and training planning processes. Indeed, the literature indicates that increased knowledge and awareness of sports technologies significantly increases individuals' intentions to use and their acceptance of technology (24, 25).

In this context, integrating theoretical and practical content on artificial intelligence and wearable technologies into sports science education programs can be considered a crucial requirement for training qualified human resources capable of adapting to the digital transformation process in the field of sports.

This research has some limitations. First, the cross-sectional design of the study limits the definitive interpretation of causal relationships between variables. Furthermore, the fact that the sample consists only of sports science students may limit the generalizability of the findings to other sports stakeholders, such as professional athletes, coaches, and individuals of varying ages and experience levels. Therefore, it is recommended that future research utilize larger and more heterogeneous samples encompassing diverse groups of athletes and professional stakeholders. In addition, the use of advanced statistical methods such as longitudinal research designs and structural equation modeling can contribute to a more comprehensive understanding of the psychological and behavioral mechanisms underlying the adoption process of artificial intelligence and wearable sports technologies.

In conclusion, this study has revealed that cognitive, affective, and behavioral attitudes toward artificial intelligence are significant determinants of attitudes toward wearable sports technologies. In particular, the strong predictive role of behavioral attitude and the mediating effects of affective and behavioral dimensions demonstrate that technology acceptance is not solely a process based on cognitive evaluations, but also a multidimensional and dynamic psychological construct encompassing emotional and behavioral components. These findings are consistent with studies examining the evolution of technology acceptance models and support the idea that cognitive, affective, and behavioral factors have a holistic impact on technology use intention and acceptance (28).

## 5 Conclusions

Accordingly, it can be said that for the effective application of artificial intelligence and wearable technologies in sports environments, not only should technological infrastructure be developed, but also education and practices aimed at increasing individuals' knowledge levels, awareness, and psychological readiness for these technologies should be encouraged.

## Data Availability

The raw data supporting the conclusions of this article will be made available by the authors, without undue reservation.
